# The Different Spatial Distribution Patterns of Nitrifying and Denitrifying Microbiome in the Biofilters of the Recirculating Aquaculture System

**DOI:** 10.3390/microorganisms13081833

**Published:** 2025-08-06

**Authors:** Wenwen Jiang, Tingting Liu, Shuting Li, Li Li, Kefeng Xu, Guodong Wang, Enmian Guo

**Affiliations:** 1 School of Marine Science and Engineering, Qingdao Agricultural University, Qingdao 266109, China; jiangww@qau.edu.cn (W.J.);; 2 Key Laboratory of Mariculture, Ministry of Education, Ocean University of China, Qingdao 266003, China; 3 Function Laboratory for Marine Fisheries Science and Food Production Processes, Qingdao National Laboratory for Marine Science and Technology, Qingdao 266235, China; 4 Marine Science Research Institute of Shandong Province, National Oceanographic Center, Qingdao 266104, China

**Keywords:** recirculating aquaculture system, biofilter, nitrifying, denitrifying, microbiome

## Abstract

In this study, the distribution patterns of the nitrifying and denitrifying microbiome in a large-scale biofilter (587.24 m^3^) in a cold freshwater recirculating aquaculture system (RAS) was investigated. Previous studies have revealed that the water quality, nitrification, and denitrification rates in the front (BFF), middle (BFM), and back (BFB) of this biofilter are different. The results showed the highest diversity of the denitrifying microbiome in the BFB, followed by BFF and BFM, whereas nitrifying microbiome diversity remained consistent across different positions. Two genera, *Nitrosomonas* and *Nitrosospira*, dominated the nitrifying microbiome, while *Pseudomonas*, *Thauera*, *Cupriavidus*, *Dechloromonas*, *Azoarcus*, and *Paracoccus* comprised the top six denitrifying genera. Principal coordinate analysis indicated a distinct spatial distribution pattern of the denitrifying microbiome but not the nitrifying microbiome. The genera *Pseudomonas* and *Dechloromonas* were the biomarkers of the BFF and BFB, respectively. Redundancy analysis showed that nitrite, nitrate, dissolved oxygen, and soluble reactive phosphorus influenced the functional microbiome distribution pattern. Network correlation analysis identified one nitrifying hub (*Nitrosospira*) in the BFF, five denitrifying hubs (*Aromatoleum*, *Dechloromonas*, *Paracoccus*, *Ruegeria*, and *Thauera*) in the BFM, and three denitrifying hubs (*Azoarcus*, *Magnetospirillum*, and *Thauera*) in the BFB. Exclusively negative correlations were found between hubs and its adjacent nodes in the BFF and BFB. This study demonstrates that habitat can shape the distribution patterns of the nitrifying and denitrifying microbiome in the biofilter of the RAS, with the BFF exhibiting greater benefits for the nitrification process.

## 1. Introduction

Because of the comprehensive management of water use, culture conditions, and waste streams, the recirculating aquaculture system has been widely used in various regions of the world [1,2]. In the RAS, high feed inputs usually result in organic waste increases. These wastes can be decomposed by microorganisms in the RAS, during which organic nitrogen is transformed into ammonia nitrogen, which is highly toxic to the cultured aquatic animals [3,4]. Thus, how to efficiently convert the toxic ammonia to non-toxic nitrogen becomes an urgent need in the RAS.

In the biofilter of the RAS, ammonia is converted to less toxic nitrate via nitrification. This process involves different functional microbiomes, where ammonia is initially oxidized to nitrite by ammonia-oxidizing bacteria (AOB), followed by nitrite oxidation to nitrate through nitrite-oxidizing bacteria (NOB) [5,6]. Additionally, the biofilter nitrogen removal process contains other processes, such as denitrification. This process usually happens in the anoxic zones of the biofilter, and performs the function of reducing nitrate to dinitrogen gas (N_2_) under an anoxic condition [7]. Consequently, revealing the characteristics of functional microbiomes in the biofilters is essential for RAS optimization.

Ammonia oxidation is the first and rate-limiting step in nitrification. Studies have reported that certain environmental factors can inhibit nitrification by altering ammonia-oxidizing microbiomes [8]. Consequently, the *amoA* gene—encoding ammonia monooxygenase—is widely used to explore the microbial nitrification mechanism across diverse environments [8,9]. The microbial denitrification is mainly represented by two genes: *nirK* (encoding the copper-containing reductase) and *nirS* (encoding the cytochrome cd1 nitrite reductase) [10,11]. Among these, *NirS* is more frequently utilized due to its higher diversity, greater abundance [12], and robust activity in different ecosystems [9,13]. In various environments, such as soil, the wastewater bioreactor, constructed wetland et al., the nitrifying microbiome encoded by the *amoA* gene and denitrifying microbiome encoded by the *nirS* gene have been extensively studied in revealing their microbial nitrogen cycle mechanism [8,9,14]. However, most studies of RAS biofilters mainly focused on the whole microbial communities by 16S rRNA sequencing [15,16]. The characteristics of the nitrifying and denitrifying microbiomes in the biofilters of the RAS, particularly their spatial distribution patterns, remain unclear, which potentially impedes RAS optimization. Furthermore, the environmental microbiome is variable, frequently shifting under varying environmental conditions. It has been reported that oxygen, organic carbon, and pH have a direct influence on the environmental microbial communities [9,17,18]. In different biofilters of the RAS, the water quality is significantly different [19]. However, information about how the functional microbiomes interact with the environmental conditions, and the relationship between the main functional microorganisms and the water quality in the biofilter of the RAS is still limited [20].

Here, a large-sized commercial biofilter (587.24 m^3^) was set up in a RAS for salmonid culture. Previous work has confirmed that the water quality and nitrification and denitrification rates are different in different positions of the biofilter. The highest nitrification and denitrification rate was found in the front and back of the biofilter, respectively [21]. But its specific microbial mechanism was still unclear. Therefore, this study further explored the nitrifying and denitrifying microbiomes at different positions of the biofilter. In contrast to previous studies, this study illustrated the specific variations in both nitrifying and denitrifying microbiomes in the biofilter of the RAS, identified key genera both in the microbial community structure and the microbial interactions, and further elucidated their relationship with water quality. This study will contribute to understanding how the nitrifying and denitrifying microbiome is distributed in the biofilter of the RAS, and its relationships with the environmental factors. The results will be facilitated for the successful management and performance of the RAS.

## 2. Material and Methods

### 2.1. RAS System Description

The recirculating aquaculture system (RAS) was located in Rizhao, Wanzefeng Co., Ltd., Rizhao, Shandong Province, China, with a low water temperature between 17 and 18 °C. The system consisted of fish-rearing tanks (FRTs), a drum filter, bio-filter (BF), and ultraviolet disinfection and oxygenation systems (Figure S1). The FRT included 26 cylindrical fiber-reinforced plastic tanks (diameter, 6 m; deep, 1.2 m). The biofilters contained six concrete tanks of the same size and the volume of the biofilter was 587.24 m^3^. The bio-medias were submerged polyethylene elastic material (0.5 mm in diameter and a specific surface area of approximately 360 m^2^/m^3^). Fish-rearing water was used to incubate the biofilters for ten days; after that, the system was running with 20 cycles one day to culture steelhead trout (*Oncorhynchus mykiss* gairdneri) in 2016.

### 2.2. Sampling Design

The biofilter included six independent concrete tanks and water flowed successively from the first to the last concrete tanks. In August, 2017, water samples were collected from the first, third, and fifth tanks of the biofilter representing the front (BFF), middle (BFM), and back (BFB) of the biofilter, respectively. In the other study, we had investigated the bacterial community succession during the start-up phase of biofilters and demonstrated the biofilters matured in two months after the incubation [22]. The results can demonstrate that the biofilm in the biofilter was mature when we sampled. The water temperature, pH, and dissolved oxygen (DO) in the biofilter were measured by a YSI portable meter (YSI Incorporated, Yellow Springs, OH, USA) on field. Water samples were collected from the surface (0.5 m below the surface), middle (half of the biofilter depth), and bottom (0.5 m above the bottom) depth of the biofilter using a plexiglass water sampler and mixed homogeneously. The collected water samples were stored in 500 mL sterile poly-propylene bottles and transported to the laboratory in Ocean University of China, Qingdao, on ice for analysis. The concentration of the total ammonia nitrogen (TAN), nitrite (NO_2_^−^-N), nitrate (NO_3_^−^-N), and soluble reactive phosphorus (SRP) was measured following the method by [23]. The particulate organic carbon (POC) was collected using pre-combusted Whatman GF/F filter membranes (pore size 0.22 μm), which were then exposed to HCl fumes for 4 h to remove the inorganic carbon. The POC was measured by an Elemental Analyzer (Elementar, Dortmund, Germany). Dissolved organic carbon (DOC) in water was measured by a total carbon analyzer (Multi N/C 2100S, AnalyticJena, Jena, Germany) after being filtered by GF/F membranes. The POC and DOC were summarized to obtain the total organic carbon (TOC) in water [24].

Five grams of biofilm samples were collected at the front, middle, and back of the biofilter, respectively. Three replicates were set for each site. The biofilm was placed in a sterile centrifuge tube and the tubes were stored at −80 °C until DNA extraction.

### 2.3. DNA Extraction and Sequencing

The total genome DNA of the biofilm samples was extracted using a Cetyl trimethyl ammonia bromide (CTAB) method according to the manufacturer’s protocol. The integrity of the DNA was tested by 1% agarose gel. The concentration of DNA was measured using NanoDrop 1000 (Thermo Scientific, Waltham, MA, USA). After that, DNA was diluted to 1 ng/μL by sterile water. Primers of *amoA*-1F/*amoA*-2R (*amoA*-1F:5′-GGGGTTTCTACTGGTGGT-3′, *amoA*-2R:3′-CCCCTCKGSAAAGCCTTCTTC-5′) were used to amplify the AOB community, and primers of Cd3aF/R3cd (Cd3aF:5′-GTSAACGTSAAGGARACSGG-3′, R3cd:5′-GASTTCGGRTGSGTCTTGA-3′) were used for amplifying the denitrifying bacteria. All PCR reactions were carried out in a 30 μL reaction system, which contained 15 μL of PCR Master Mix (New England Biolabs, Ipswich, MA, USA), 0.2 μM of forward and reverse primers, and about 10 ng of DNA templates. The PCR condition was set as initial denaturation at 98 °C for 1 min, followed by 30 cycles of denaturation at 98 °C for 10 s, annealing at 50 °C for 30 s, elongation at 72 °C for 60 s, and final 72 °C for 5 min. To qualify the PCR products, mix the same volume of both 1× loading buffer (contained SYB green), and PCR products were mixed and operated electrophoresis on 2% agarose gel. The amplicons obtained were then purified using a GeneJET Gel Extraction Kit (Thermal Scientific, USA).

Sequencing libraries were generated using the NEB Next^®^Ultra™DNA Library Prep Kit for Illumina (NEB, Ipswich, MA, USA), following the manufacturer’s protocol, and were assessed on the Qubit@ 2.0 Fluorometer (Thermo Scientific, USA) and Agilent Bioanalyzer 2100 system. Lastly, it was sequenced by the Illumina MiSeq platform.

### 2.4. Sequencing Data Processing

Paired-end reads from the original DNA fragments were merged using FLASH (v. 1.2.7). Low-quality sequences were filtered by Trimmomatic (v. 0.33). Chimeric sequences were identified and removed using UCHIME (v. 4.2). A similarity cutoff of >97% was used to assign the same OTUs by QIIME (v. 1.9.0) when only DNA sequence data were available. The OTUs were annotated against the SILVA 135 database.

### 2.5. Statistical Analysis

All of the raw sequences were deposited in the NCBI Sequence Read Archive (SRA) database under the accession number of PRJNA741549.

The α-diversity indices were analyzed by QIIME (v. 1.9.1). Principal coordinate analysis (PCoA) of Bray–Curtis dissimilarity was performed using a vegan package in the R platform (v. 3.6.2). Biomarkers of functional taxa at different positions were identified using the effect size of linear discriminant analysis (LEfSe) (http://huttenhower.sph.harvard.edu/lefse/) (accessed on 26 December 2021), and the threshold of LDA scores was set as 3.5. Redundancy analysis (RDA) was performed using the vegan package in the R platform (v. 3.6.2). Before RDA analysis, environmental factors with variance inflation factors (VIF) <10 were selected to exclude the collinearity. The microbial co-occurrence network was inferred using a plugin of CoNet (v. 1.1.1) in Cytoscape (v. 3.7.2). Specifically, four similarity measurements (Pearson and Spearman correlations; Bray–Curtis and Kullback–Leibler non-parametric dissimilarities) were used to reveal the 50 top and bottom results. For each measurement, 100 renormalized and 100 bootstrap scores were calculated. Then, Brown and Benjamin–Hochberg methods were further used to avoid the false positive results. In the microbial co-occurrence network, taxa with the highest connectivity were considered as the network hub. Then, Pearson correlation between the network hubs and environmental variables was further performed and visualized using a corrplot package in the R platform (v. 3.6.2).

The mean ± standard deviation (SD) was presented for the different parameters measured. The normality and homogeneity of variance of data were firstly checked by the Kolmogorov–Smirnov test and Levene’s test, respectively. If data fit the assumption, one-way analysis of variance (ANOVA) followed by a multiple comparison test (Turkey) was applied to test for differences of water quality parameters and α-diversity indices of bacterial communities in different positions of biofilter. Otherwise, data were log-transformed or performed the non-parametric Kruskal–Wallis test. Differences were considered significant at *p* < 0.05. The above-mentioned statistical analysis was performed in SPSS statistic 20 (IBM, New York, NY, USA).

## 3. Results

### 3.1. Water Quality Characteristics in Different Positions of the Biofilter

The water quality in three positions of the biofilter (BFF, BFM, and BFB) was presented in Table 1. The results showed that there were significant differences in total ammonia nitrogen (TAN), nitrite (NO_2_^−^-N), nitrate (NO_3_^−^-N), pH, and total organic carbon (TOC) among sampling sites (*p* < 0.05), while temperature (T), dissolved oxygen (DO), and solubility reactive phosphorus (SRP) presented no significant differences (*p* > 0.05). Specifically, concentrations of TAN were higher in BFF and BFM than BFB, with a value of 0.16, 0.19, and 0.11 mg L^−1^, respectively. The highest NO_2_^−^-N concentration was presented in BFM, with a value of 0.21 mg L^−1^ (*p* < 0.05). The NO_3_^−^-N concentration presented a different trend, with the highest value in BFB (10.57 mg L^−1^), followed by BFF and BFM. The TOC concentration in BFB presented the lowest, with a value of 13.74 mg L^−1^, while the sample in BFF had the highest, with a value of 34.54 mg L^−1^, followed by BFM, with a value of 24.71 mg L^−1^. The pH in BFB was the lowest, with a value of 7.31.

### 3.2. Microbial Diversity Characteristics of Functional Microbiomes in Different Positions of the Biofilter

The alpha diversity including Coverage, Chao1, Shannon, and Simpson of nitrifying and denitrifying microbiomes at different positions of the biofilter is listed in Table 2. The microbiome sequencing data obtained with a coverage of 99%, indicating good microbiome information, was obtained. The index of Chao1 indicates the richness of the microbiome, and the indices of Shannon and Simpson indicate both evenness and diversity. In the nitrifying microbiome, the Chao1, Shannon, and Simpson did not present significant differences, indicating that there were no significant differences both in the diversity and richness of the nitrifying microbiome in different locations of the biofilter (*p* > 0.05). In the denitrifying microbiome, there was also no significant differences in Chao1 in different positions (*p* > 0.05), indicating no differences in richness. But both Shannon and Simpson presented the significantly highest value in BFB, indicating that the denitrifying microbiome in BFB had the largest diversity, followed by BFF and BFM.

**Figure 1:**
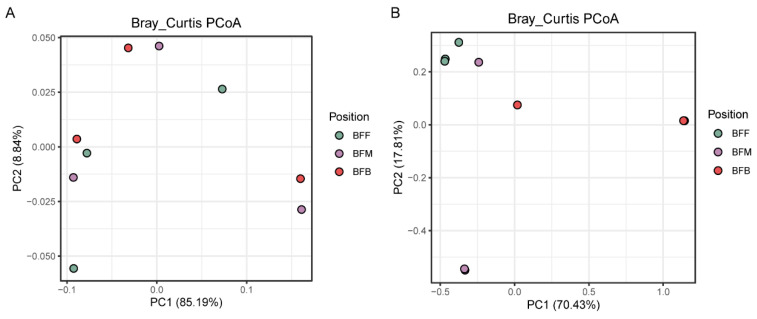
PCoA ordination of nitrifying microbiome (**A**) and denitrifying microbiome (**B**) based on Bray−Curtis similarities profiles in biofilm samples from different positions of the biofilter. BFF, BFM, and BFB indicated the front, middle, and back of the biofilter.

Principal coordinate analysis (PCoA) based on Bray–Curtis distance could present the differences in the microbial community among different positions (Figure 1). The result showed that the nitrifying microbiome from different sampling positions did not separate well. However, the samples from BFF and BFB could be separated based on the diversity of the denitrifying microbiome, suggesting a remarkable difference between the two sites.

### 3.3. Functional Community Structure in Different Positions of the Biofilter

**Figure 2:**
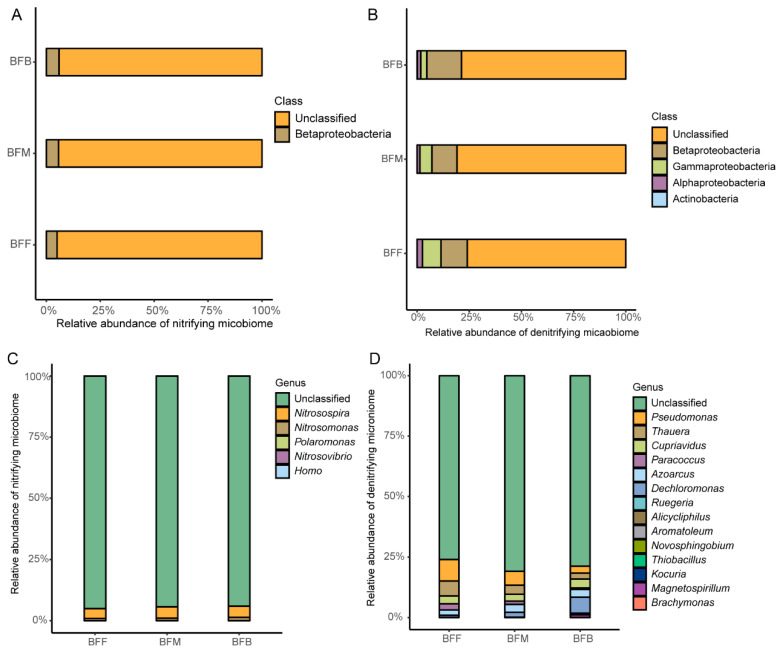
The relative abundance of nitrifying microbiome and denitrifying microbiome at class level (**A**,**B**) and genus level (**C**,**D**). BFF, BFM, and BFB indicated the front, middle, and back of the biofilter.

The community structures of both the nitrifying and denitrifying microbiome in different positions of the biofilter were different (Figure 2). In the nitrifying microbiome, the dominant bacteria at the class level were Betaproteobacteria, which belongs to the phylum Proteobacteria (Figure 2A). The highest relative abundance of Betaproteobacteria was presented in BFB, with a value of 5.89%, followed by BFF and BFM. At the genus level, the most abundant genera were affiliated with *Nitrosomonas* and *Nitrosospira* (Figure 2C). The *Nitrosospira* occupied a relative abundance of 4.06% in BFF, 4.71% in BFM, and 4.57% in BFB, while *Nitrosomonas* occupied 0.90%, 1.01%, and 1.32% in BFF, BFM, and BFB, respectively.

In the denitrifying microbiome, the classes of Betaproteobacteria, Gammaproteobacteria, and Alphaproteobacteria were identified as the dominant taxa (Figure 2B). The Betaproteobaceria class was the dominant class observed at all sampling sites and with the relative abundances of 12.60% at BFF, 12.06% at BFM, and 16.63% at BFB. The top six genera (average abundance) defined were *Pseudomonas*, *Thauera*, *Cupriavidus*, *Dechloromonas*, *Azoarcus*, and *Paracoccus* (Figure 2D). The abundance of *Pseudomonas*, *Thauera*, and *Paracoccus* decreased from the front to the back of the biofilter. The relative abundance of *Pseudomonas* in BFF, BFM, and BFB was 8.87%, 5.71%, and 2.87%, respectively. The relative abundance of *Thauera* was 6.27% at BFF, 3.76% at BFM, and 2.48% at BFB. And the relative abundance of *Paracoccus* in the front, middle, and back of the biofilter was 2.54%, 1.38%, and 0.60%, respectively. But the *Dechloromonas* increased from the front to the back of the biofilter. The relative abundance of *Dechloromonas* in BFF, BFM, and BFB was 0.90%, 1.98%, and 6.61%, respectively.

### 3.4. Functional Biomarkers in Different Positions of the Biofilter

**Figure 3:**
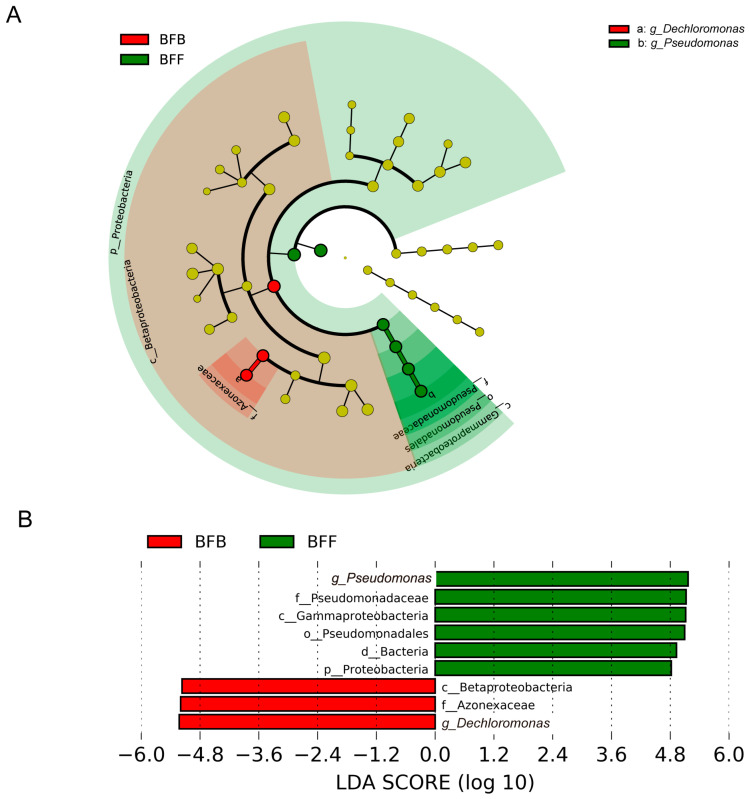
Cladogram indicating the phylogenetic distribution of bacterial lineages at different positions of the biofilter (**A**). The labels are shown at the genus level only. The six rings of the cladogram stand for domain (innermost) phylum, class, order, family, and genus. (**B**) LDA score of the three systems with a threshold value of 3.5. BFF, BFM, and BFB indicated the front, middle, and back of the biofilter, respectively.

The effect size of linear discriminant analysis (LEfSe) was used to identify different abundant taxa (biomarkers) at different positions of the biofilter. As shown in Figure 3, there were nine bacterial clades showing significant differences between the front and back of the biofilter when the LDA threshold was 3.5. Six identified bacteria clades were enriched in BFF, while three bacteria showed greater abundance in BFB. At the phylum level, the Proteobacteria (LDA score of 4.8) dominated in BFF. At the class level, Gammaproteobacteria (LDA score of 5.1) and Betaproteobacteria (LDA score of 5.2) were enriched in BFF and BFB, respectively. The genera *Pseudomonas* (LDA score of 5.2) and *Dechloromonas* (LDA score of 5.2) had higher abundance in the front and back of the biofilter, respectively (Figure 3). No significant biomarkers were identified in the middle of the biofilter (BFM).

### 3.5. Microbial Network Analysis in Different Positions of the Biofilter

**Figure 4:**
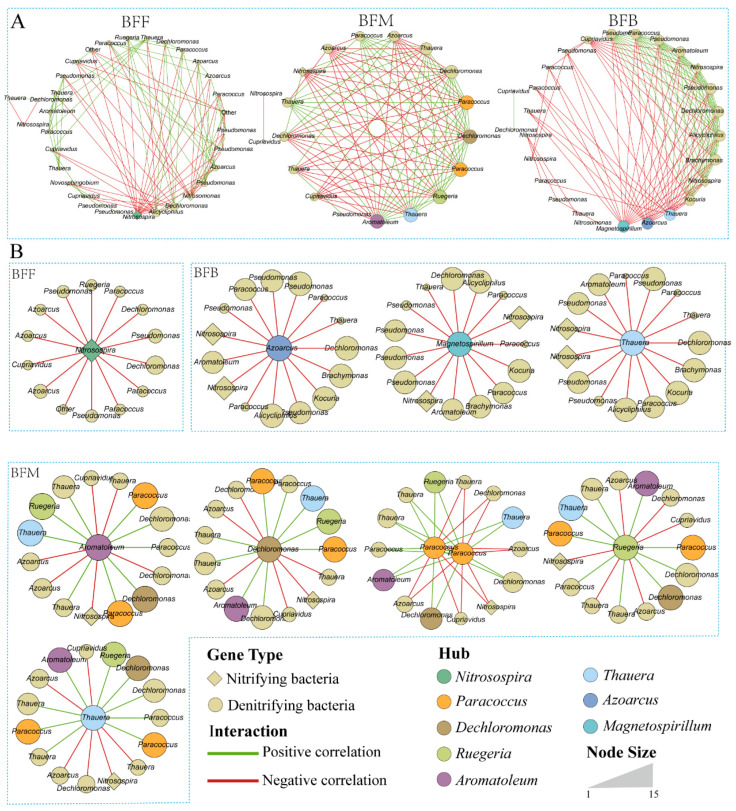
Network analysis on the relationship among denitrifying and denitrifying microbiomes (**A**), and correlations of the hubs (**B**) at different sampling positions. BFF, BFM, and BFB indicated the front, middle, and back of the biofilter, respectively.

Network analysis was used to examine the relationship among species of nitrifying and denitrifying microbiomes. According to the average number of neighbors, the complexity of network in the BFM was the highest followed by BFB and BFF (Figure 4A). Network hubs were defined as taxa exhibiting the highest connectivity (most adjacent nodes). The number of hubs in BFM was the highest followed by BFB and BFF. The correlations between hubs and other taxa in the microbial network at different sampling positions were further extracted (Figure 4B). The hub of BFF was *Nitrosospira*, which had a negative correlation with all its adjacent nodes. In the back of the biofilter, three denitrification bacteria hubs, *Azoarcus*, *Magnetospirillum*, and *Thauera*, were identified. The relationships between hubs and their adjacent nodes were also mainly negative. Five hubs (*Aromatoleum*, *Dechloromonas*, *Paracoccus*, *Ruegeria*, and *Thauera*) were found in BFM, and all these hubs were denitrification bacteria. Notably, these hubs had both positive and negative relationships with adjacent nodes.

### 3.6. Effect of Environmental Conditions on Distribution Pattern of the Functional Microbiome

**Figure 5:**
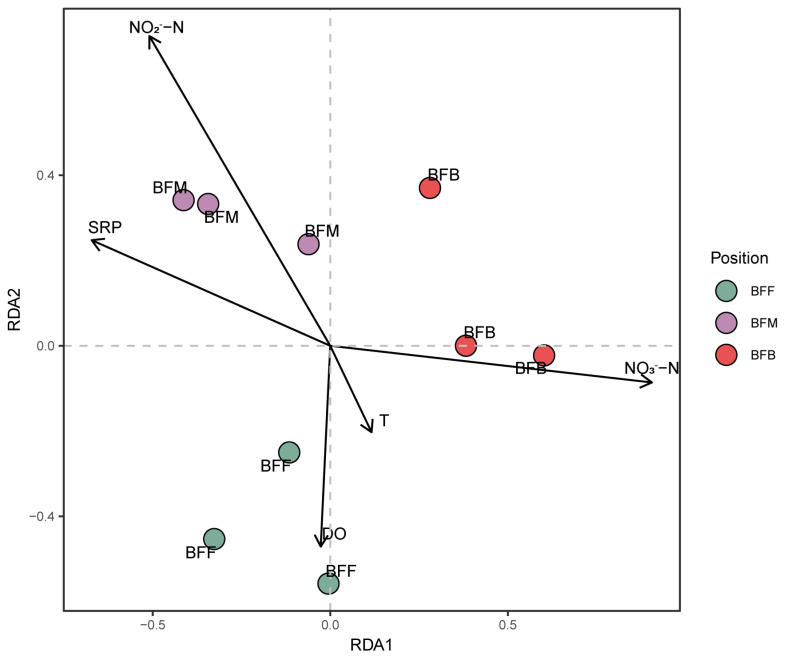
Redundancy analysis (RDA) on the relationship between abiotic environment parameters and denitrifying and denitrifying microbiomes at different sampling positions. BFF, BFM, and BFB indicated the front, middle, and back of the biofilter, respectively. The arrows represent the effect of environment conditions on the nitrifying and denitrifying microbiomes in the biofilters.

Redundancy analysis was used to explore the effect of environment conditions on the nitrifying and denitrifying microbiomes in the biofilters. The samples collected from different positions of the biofilter separated well. The RDA analysis fits well (F = 8.39, *p* < 0.05), and the first two principal RDA axes could totally explain 50.07% and 12.92% of the variances in the functional microbiome (Figure 5). Eight environmental factors (T, DO, pH, TAN, NO_2_^−^-N, NO_3_^−^-N, SRP, and TOC) were first selected by a collinearar analysis, where the TAN, pH, and TOC were excluded due to their collinearity. The NO_2_^−^-N and NO_3_^−^-N were the top two significant variables, which might play critical roles in reshaping both the nitrifying and denitrifying microbiome in the biofilter. The NO_2_^−^-N positively affected the structure of the functional microbiome in the BFM. The structure of function genes in BFB was positively affected by the NO_3_^−^-N concentration in the biofilter. Additionally, DO might positively affect the functional microbiome in the BFF. Meanwhile, temperature (T) did not pose much weight on the separation of the distribution of the function genes at different sampling sites.

**Figure 6:**
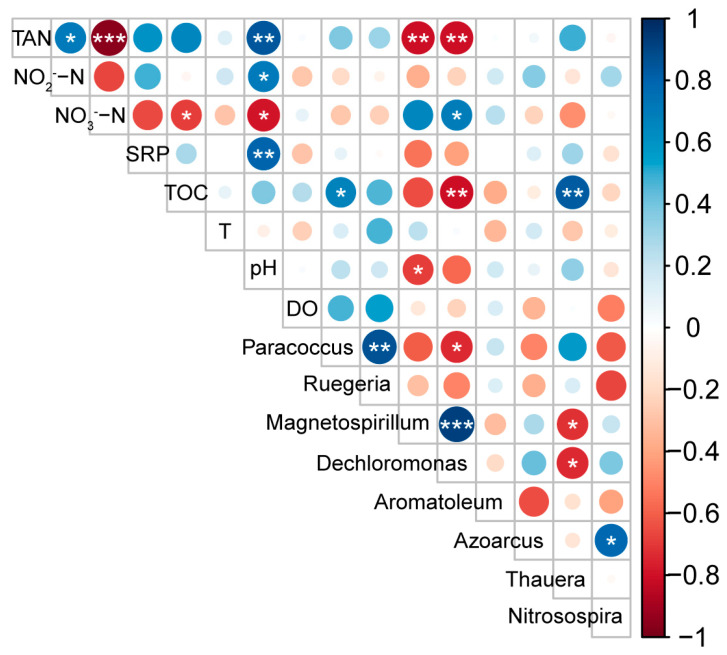
Pearson correlation analysis on the relationship between environmental factors and the hubs selected by network analysis. * indicates significantly correlation with *p* < 0.05, ** indicates significant correlation with *p* < 0.01 and *** indicates significant correlation with *p* < 0.001.

Pearson correlation analysis was further used to explore the relationship of environment factors and their effects on the hubs (Figure 6). The results showed that the hubs of *Magnetospirillum* and *Dechloromonas* were negatively correlated with TAN, while *Dechloromonas* was positively correlated with NO_3_^−^-N. The TOC was positively correlated with the hubs of *Thauera* and *Paracoccus*, but negatively correlated with *Dechloromonas*. *Magnetospirillum* was negatively correlated with pH.

## 4. Discussion

### 4.1. Diversity of Nitrifying and Denitrifying Microbiome in RAS

The *Nitrosomonas* and *Nitrosospira* were the most important genera in the nitrifying microbiome in the present biofilter. These taxa were commonly detected in industrial and domestic wastewater treatments plants, as well as RAS biofilters [6,25,26,27]. The *Nitrosospira* presented a higher proportion than *Nitrosomonas* in this study. Previous findings reported that the *Nitrosomonas* spp. generally dominated *Nitrosospira* spp. in the environment, such as soil and wastewater treatments plants, because of their superior growth rate [25,26,28]. The two genera were present in equal amounts in granular sludge, whereas *Nitrosomonas* was the dominant genus in flocculent sludge [29]. The two genera preferred different environmental conditions. Environmental factors including organic load, temperature, and DO were reported to modulate their competitive balance [5,9,29]. The reason for the higher percentage of *Nitrosospira* over *Nitrosomonas* in the present study is not clear. The mutual interaction between the two genera in the biofilter of the RAS needs further investigation. Besides the genera of *Nitrosomonas* and *Nitrosospira*, *Nitrosococcus* also belongs to the class of Gamaproteobacteria, which are the lithotrophic ammonia oxidizers found in the biofilter of the RAS [30,31]. However, no *Nitrosococcus* spp. were detected in our study. The reason for this is that the RAS in this study is freshwater. The genus *Nitrosococcus* has two described species, *N. oceani* and *N. halophilum*, which are obligately halophilic [30].

The denitrification in the biofilter was conducted by a diverse, versatile population including Betaproteobacteria, Alphaproteobacteria, and Gamaproteobacteria. This result is in accordance with previous findings, which also reported the presence of a diverse bacteria population in activated sludge [32]. The *Thauera*, *Azoarcus*, *Paracoccus*, and *Dechloromonas* were all denitrifiers found in the bioreactor treating landfill leachate [9]. The *Azoarcus* was the primary nitrogen converter in the landfill bioreactor [33]. The *Cupriavidus* is one of the main genera of denitrifiers identified in soil [13]. The genera *Pseudomonas* and *Thauera* had the highest average abundance in the biofilter of the present study. *Pseudomonas* and *Thauera* were also the main denitrifying bacteria genera in the sediment of Hangzhou Bay [34]. The heterotrophic denitrifiers *Pseudomonas* spp. appeared to be the most abundant Gamaproteobacteria in marine RAS nitrification filters [3,31]. This genus includes biofilm-forming species, which could colonize a wide range of micro-niches [6].

### 4.2. Network Characteristics of the Different Functional Microbiomes

Network correlation analysis is a classical method for analyzing the taxa interrelationships in microbial communities [35]. The hub of BFF was *Nitrosospira*, which is an ammonia-oxidizing bacteria. Ammonia is converted to a less hazardous nitrate through nitrification in the biofilter of the RAS [31]. When the water flowed into the biofilter in the present study, nitrification first happened in the front of the biofilter. Our earlier study demonstrated that the nitrification rate was the highest at the front of the biofilter [36]. In BFM and BFB, all identified hubs were denitrifying bacteria. Specifically, three denitrifying bacteria hubs, *Azoarcus*, *Magnetospirillum*, and *Thauera*, were identified within BFB. Network analysis indicated that *Magnetospirillum* was significantly associated with 12 other denitrifying genera and played a particularly predominant role in promoting or inhibiting the growth of other bacterial genera in a novel DAS-NUA biofilter used for groundwater nitrate treatment [35]. Additionally, the genus *Thauera* was identified as a hub both in BFM and BFB, suggesting its critical role in the ecological network of denitrifying bacteria.

In the constructed network, mutualism or commensalism represents positive interactions of microbial species, whereas competition, predation, and amensalism represent negative relationships [22,37]. In addition, competitions rather than predations were regarded as the main negative relationships among bacterial species [38]. The hubs in BFM exhibited both positive and negative relationships with their adjacent nodes, while the hubs in BFF and BFB presented a negative correlation with all of their neighboring nodes. When the biofilter matured, a decrease in positive relationships and increase in negative relationships were observed in the biofilter of the present RAS in an earlier study [22]. Jiang [22] also demonstrated that the diversity of bacterial communities in the biofilter increased when it matured. As different species reflect differently to environmental changes, the increasing biodiversity ensures ecosystems against the functioning decline, and then the environment changes [39]. The denitrifying bacteria had the largest diversity in BFB, followed by BFF and BFM, indicating that the BFB had a more mature denitrifying community than the BFM. The microbial community in the BFM was probably still unstable, which could also be verified by the complexity of the network. The BFM had the highest complexity of the microbial network. A previous study reported that after environment perturbations (emulsified vegetable oil amendment), the complexity of microbial connections in groundwater increased first and then decreased at a later state [38].

### 4.3. Environmental Factors Effect on the Distribution Pattern of Functional Microbiome in the Biofilter

Water quality exhibited distinct spatial variation across different positions of the biofilter. The front of the biofilter had a higher DO, pH, TAN, and TOC, and lower NO_3_^–^-N than the back of the biofilter. This spatial gradient aligned with the results from this biofilter over the past several months [36]. One of the main functions of the biofilter in the RAS is to convert the toxic TAN into a less toxic nitrate. The process happens in two reactions. In the first reaction, TAN is converted to NO_2_^−^-N (NH_4_^+^ + 1.5 O_2_ → NO_2_^−^ + 2H^+^ + H_2_O). In the second reaction, NO_2_^−^-N is converted to NO_3_^−^-N (NO_2_^−^ + 0.5 O_2_ → NO_3_^−^) [33]. This process consumes O_2_ and reduces the pH. Due to the occurnence of nitrification, the DO, pH, and TAN were decreased from the front to the back of the biofilter in this study. Conversely, the NO_3_^−^-N concentration showed the opposite trend, presenting the highest level at the back of the biofilter. This result was consistent with the diversity of denitrifiers, which was highest at the back of the biofilter, indicating that NO_3_^−^-N was critical in reshaping the denitrifiers of the biofilter. When the water flows from the front to the back, the bio-medias in the biofilter intercepted organic materials in the water, causing the decrease in the TOC from the front to the back of the biofilter.

The structure of denitrifiers differed at different positions of the biofilter. Previous studies also reported that the denitrifying bacteria community was affected by land use, fertilization, and substrate in the environment such as soil or activated sludge from nutrient removal plants [13,32]. In this study, denitrifiers presented the highest abundance and diversity at the back of the biofilter, but were lower in the middle than at the front. However, the abundance of some denitrifiers did not show many differences among different sampling positions, such as *Azoarcus* and *Cupriavidus*. The abundance of *Pseudomonas*, *Thauera*, and *Paracoccus* decreased, and *Dechloromonas* increased from the front to the back of the biofilter. This is related to different water quality characteristics at different positions. The TOC was highest at the front of the biofilter. Pearson correlation analysis of the present study indicated that the TOC was positively correlated with *Thauera* and *Paracoccus* and negatively correlated with *Dechloromonas*. This is consistent with the findings of a previous study. In a biofloc-based shrimp culture system, the abundance of the denitrification bacteria *Paracocccus* increased in the treatments of added carbon sources, and the abundance of this genus was positively correlated with the TOC [40]. The *Thauera*-related *nirS* gene was reported to be predominant under high organic load in the bioreactor treating landfill leachate, while to a lower extent, the *Dechloromonas* is affected [9]. *Thauera*, which is frequently detected in wastewater treatment bioreactors, is capable of both denitrification and organic compound biodegradation [28]. Moreover, *Thauera* has been reported to proliferate in the treatments of ammonium-rich wastewater [28]. In this study, the culturing water in BFF had a higher TAN concentration than in BFB.

Besides the highest abundance, the genus *Dechloromonas* was selected as a biomarker in the BFB by LEfSe (Figure 3). The abundance of *Dechloromonas* was negatively correlated with TAN and positively correlated with NO_3_^−^-N. This is supported by the finding of Xu [35], who reported there was a positive effect of NO_3_^−^-N and negative effects of TAN on the denitrification gene in a novel biofilter which was used for groundwater nitrate treatment. These two environmental factors were also reported to play important roles in shaping denitrification community structure in a constructed wetland treating polluted river water [41].

Besides the highest abundance, the genus *Pseudomonas* was selected as a biomarker in the BFF by LEfSe (Figure 3). The results of RDA indicated that the structure of the biomarkers in BFF was positively related to DO. The TOC was excluded from RDA analysis due to collinearity. Pearson correlation analysis indicated that the TOC was positively correlated with DO. The *Pseudomonas* was a heterotrophic denitrifier, which favored a high C/N [3,31]. The community structure of AOB was reported to be affected by various environment factors such as pH, ammonia substrate, and organic matter [42]. As indicated by PCoA, no shifts of structure in the AOB community were found in the biofilter of the present study. This might be because the present RAS is already mature and the abiotic environments are relatively stable.

## 5. Conclusions

The nitrifying and denitrifying microbiome distribution patterns in the biofilter were investigated. The nitrifying microbiome did not present significant variations in the biofilter, while the denitrifying microbiome presented more diversity and abundance at the back of the biofilter. This distribution patterns were closely related with the water quality. The significantly different functional biomarkers were only presented in the front and back of the biofilter but not in the middle. But the complexity of the functional microbial network in the middle of the biofilter was higher than that of the front and back position. The hubs of the microbial network at the front of the biofilter were dominated by nitrifiers, while the middle and back were mainly dominated by denitrifiers. The BFF was more beneficial for the nitrification process, while the BFB was more beneficial for the denitrification process.

## Figures and Tables

**Table 1:** Water quality at different positions of the biofilter during the experiment.

Parameters	T/℃	pH	DO (mg·L^−1^)	TAN (mg·L^−1^)	NO_2_^−^-N (mg·L^−1^)	NO_3_^−^-N (mg·L^−1^)	SRP (mg·L^−1^)	TOC (mg·L^−1^)
BFF	17.50 ± 0.10 ^a^	7.35 ± 0.00 ^ab^	7.77 ± 0.64 ^a^	0.16 ± 0.00 ^b^	0.11 ± 0.00 ^b^	7.69 ± 0.15 ^b^	0.08 ± 0.00 ^a^	34.54 ± 0.07 ^a^
BFM	17.53 ± 0.12 ^a^	7.40 ± 0.00 ^a^	7.38 ± 0.55 ^a^	0.19 ± 0.00 ^a^	0.21 ± 0.00 ^a^	6.43 ± 0.60 ^c^	0.09 ± 0.00 ^a^	24.71 ± 2.17 ^b^
BFB	17.47 ± 0.15 ^a^	7.31 ± 0.06 ^b^	7.48 ± 0.17 ^a^	0.11 ± 0.01 ^c^	0.12 ± 0.01 ^b^	10.57 ± 0.37 ^a^	0.08 ± 0.00 ^a^	13.74 ± 0.51 ^c^

Note: The data in the table is mean ± standard deviation, *n* = 3. BFF, BFM, and BFB are three sampling positions of this study. Different lowercase letters denote significant differences among sampling positions at *p* < 0.05 based on one-way ANOVA followed by multiple comparison test.

**Table 2:** α-diversity indices of bacterial communities in different positions of biofilter in recirculating aquaculture systems.

Functional Microbiome	Sample	OTUs	Chao1	Shannon	Simpson	Coverage
Nitrifying	BFF	20 ^a^	21 ^a^	0.99 ^a^	0.60 ^a^	0.99
	BFM	22 ^a^	22 ^a^	1.09 ^a^	0.56 ^a^	0.99
	BFB	22 ^a^	25 ^a^	1.06 ^a^	0.57 ^a^	0.99
Denitrifying	BFF	189 ^a^	193 ^a^	3.50 ^b^	0.07 ^a^	0.99
	BFM	94 ^a^	96 ^a^	3.28 ^c^	0.08 ^a^	0.99
	BFB	142 ^a^	144 ^a^	4.23 ^a^	0.03 ^b^	0.99

Note: BFF, BFM, and BFB are three sampling positions of this study. Different lowercase letters denote significant differences of the nitrifying or denitrifying microbiome among sampling positions at *p* < 0.05 based on one-way ANOVA followed by multiple comparison test.

## Data Availability

The original contributions presented in this study are included in the article. Further inquiries can be directed to the corresponding authors.

## Supplementary Materials

The following supporting information can be downloaded at: https://www.mdpi.com/article/10.3390/microorganisms13081833/s1, Figure S1: Water treatment process of recirculating aquaculture system.
