# Supplementary material for: The Different Spatial Distribution Patterns of Nitrifying and Denitrifying Microbiome in the Biofilters of the Recirculating Aquaculture System
Source: Microorganisms. 2025 Aug 6;13(8):1833. doi: 10.3390/microorganisms13081833 (PMC12388831; doi:10.3390/microorganisms13081833)
Supplement: Supplementary file 1 [file microorganisms-13-01833-s001.zip › microorganisms-3751555-supplementary.pdf]

Supplementary materials:

The Different Spatial Distribution Patterns of Nitrifying and Denitrifying Microbiome in the Biofilters of the Recirculating Aquaculture System

Wenwen Jiang [1,2], Tingting Liu [2], Shuting Li [2], Li Li [2,3], Kefeng Xu [4,*], Guodong Wang [1,*] and Enmian Guo [1]

[1] School of Marine Science and Engineering, Qingdao Agricultural University, Qingdao 266109, China; jiangww@qau.edu.cn (W.J.); emguo2013@126.com (E.G.)
[2] Key Laboratory of Mariculture, Ministry of Education, Ocean University of China, Qingdao 266003, China; 17853252219@163.com (T.L.); lishuting@stu.ouc.edu.cn (S.L.); l_li@ouc.edu.cn (L.L.)
[3] Function Laboratory for Marine Fisheries Science and Food Production Processes, Qingdao National Laboratory for Marine Science and Technology, Qingdao 266235, China
[4] Marine Science Research Institute of Shandong Province, National Oceanographic Center, Qingdao, 266104, China
* Co-correspondence: xukefeng@shandong.cn (K.X.), wgd05190729@qau.edu.cn (G.W.); Tel.: +86-532-8901-6521 (K.X.);Tel.: +86-532-8655-0511 (G.W.)

Running Title: Nitrifying and denitrifying microbiome spatial distribution pattern in biofilters

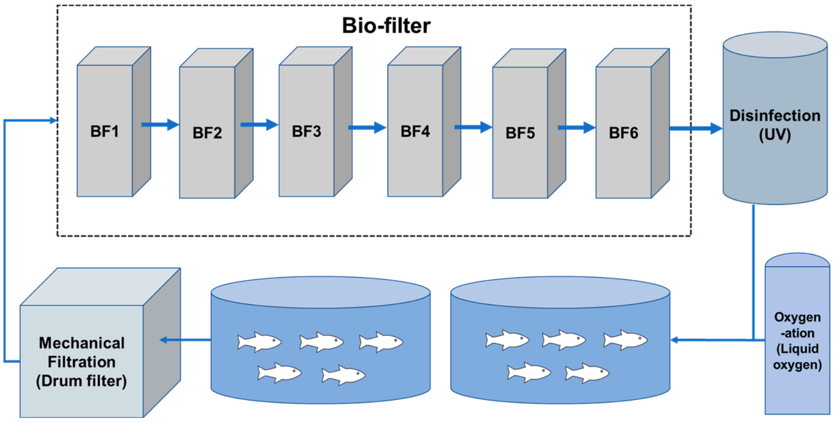

Figure S1 Water treatment process of recirculating aquaculture system.
